# A New Angle on Object-Background Effects in Vection

**DOI:** 10.1177/2041669516631695

**Published:** 2016-02-29

**Authors:** Juno Kim, Michael T. T. Tran

**Affiliations:** School of Optometry and Vision Science, University of New South Wales, Sydney, NSW, Australia

**Keywords:** self-motion perception, vection, diffuse, specular, reflectance, vision

## Abstract

We considered whether optic flow generated by 3D relief of a foreground surface might influence visually-mediated self-motion perception (vection). We generated background motion consistent with self-rotation, and a foreground object with bumpy relief was either rotated with the observer (ego-centric) or fixed in world coordinates (world-centric). We found that vection strength ratings were greater in conditions with world-centric retinal motion of the foreground object, despite generating flow that was opposite to background motion. This effect was explained by observer judgments of the axis self-rotation in depth; whereas ego-centric flow generated experiences of more on-axis self-rotation, world-centric flow generated experiences of centrifugal rotation around the foreground object. These data suggest that foreground object motion can increase the perception of self-motion generated by optic flow, even when they reduce net retinal motion coherence and promote conditions for multisensory conflict. This finding supports the view that self-motion perception depends on mid-level representations of whole-scene motion.

The primary visual stimulus for self-motion perception is “optic flow,” which is generated by the light reflected by the surfaces of objects that move *relative* to the observer (Gibson, 1950). The idea that this motion is “relative” implies that it is generated by either object or observer. The only way for the brain to differentiate between these different causes is through the visual processing of some constraints that might be diagnostic of one type of motion over the other. In some situations, the visual motion is entirely consistent with physical self-motion, and the perceptual result is the experience of compelling illusions of self-motion when completely stationary, known as “vection.”

The power of vection illusions has attracted extensive research, culminating in the strong assertion that vection critically depends on motion of some inferred background in the scene (e.g., Brandt et al., 1975; Kitazaki & Sato, 2003; Ohmi & Howard, 1988; Ohmi, Howard, & Landolt, 1987; Seno, Ito, & Sunaga, 2009). Brandt et al. (1975) found that circular vection was enhanced by stationary objects presented in the foreground, but inhibited when the same objects were situated (perceptually) behind the moving background. Ohmi et al. (1987) obtained similar findings, and Ohmi and Howard (1988) extended the effect to linear vection in depth generated by looming optic flow; a stationary pattern facilitated vection when perceived in the foreground, but suppressed vection when perceived behind the looming optic flow pattern. Kitazaki and Sato (2003) found that two transparent motions simulating upward/downward linear motion generated a perceived direction of self-motion that was opposite to the direction of the flow component perceived as farther in depth. Seno et al. (2009) proposed these effects support the hypothesis that vection is driven by motion perceived in the background and not the foreground.

Contrary to the view that vection is determined solely by perceived background motion, evidence suggests that the perceived foreground can also influence vection. Nakamura and Shimojo (1999) presented observers with two large transparent linear motions separated in depth by disparity. They found that not only did the background motion enhance linear vection, but this vection was further increased in strength when slow foreground motion occurred in the opposite direction.

Are there any plausible generative constraints on image motion(s) that might allow foreground properties of the environment to enhance vection? Observer motion relative to a curved stationary foreground object is one ecologically valid way to generate opposing directions of foreground and background motion that might increase vection. We put this to the test using realistic real-time rendering in a virtual environment viewed through a stereo head-mounted display (see Methods section). Realistic computer graphics was used to ensure the vivid appearance of a continuous foreground surface. This also ensured that all the foreshortening cues available in real-life viewing situations were present in the display. We used global illumination mapping to immerse our 16 observers inside the Uffizi Gallery of Florence Italy for up to 15 min. This provided a nice break from reality for hard working undergraduate observers who were naïve to the purposes of the current experimental protocol approved by the Human Research Ethics Advisory Panel at the University of New South Wales.

The observer’s point of view was simulated a short distance in front of the bumpy object in front of them at the center of the scene (Figure 1). There were two independent variables: fixation type and type of object motion, both with two levels; Fixation was either with free viewing or with central fixation of a red spot situated just in front of the surface at no closer than 12.5% of its maximum radius. For object motion conditions, the surrounding scene always rotated around the observer, simulating *en bloc* yaw rotation of the whole body. Whereas in two of the conditions, the central object rotated in the same direction as the scene (world-centric), the other two conditions maintained the object’s stationary orientation, holding constant the retinal image it generated (ego-centric). Note how in world-centric conditions the motion of the object’s foreground relief is right to left—the opposite of that generated by motion of the background (Figure 1(b)).

**Figure 1.**
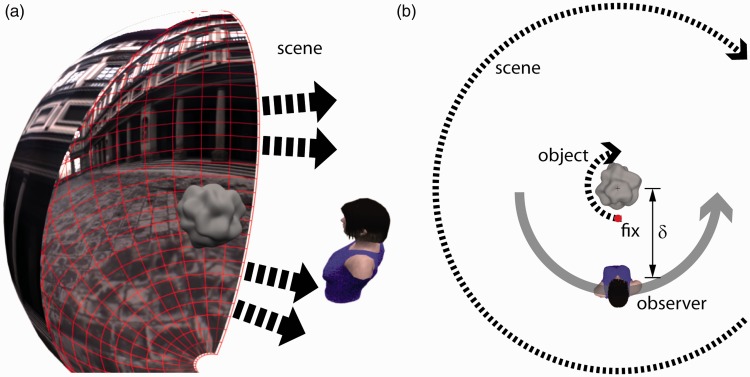
The arrangement of the simulated environment. (a) Schematic showing the organization of the background scene and foreground object relative to the observer. (b) Overview of simulated rotations of object and scene. Dashed arrows show direction of simulated physical rotation. Solid gray arrow shows the path of an observer’s perceived centrifugal orbit (rotation and translation) in the world-relief conditions. Note diagrams not to scale.

Figure 2(a) shows the results of observer estimates on their perceived axis of rotation during 10 s viewing trials of each simulation condition. A value of zero corresponds to on-center axis rotations, like spinning on a swivel chair. A value of 1 corresponds to off-center axis rotations, where the axis of rotation is situated at the center of the bumpy object, like being spun in a centrifuge. A two-way ANOVA (analysis of variance) showed no significant main effect of fixation type, *F*(1, 15) = 3.22, *p* = .093. However, there was a significant main effect of relief motion, *F*(1, 15) = 20.62, *p* < .0005. No interaction was found, *F*(1, 15) = 2.09, *p* = .17.

**Figure 2.**
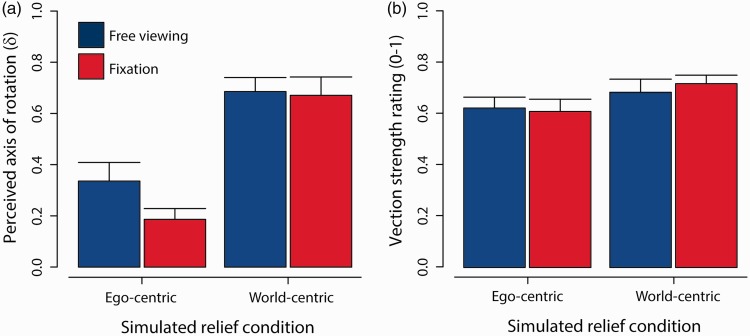
Perceptual estimates of self-motion. (a) Mean location of perceived axis of rotation in depth (δ) for conditions with (red) or without fixation (blue) during presentation of ego-centric and world-centric relief. Larger values correspond to increasingly centrifugal rotation, whereas lower values correspond to increasingly on-center axis rotation. (b) Mean vection strength ratings for the same simulation and viewing conditions. Error bars show standard errors of the mean.

These results show that foreground relief motion has a profound effect on the perceived axis of rotation; compared with more on-center yaw rotation experienced during ego-centric relief motion, observers perceive more centrifugal self-motion when viewing world-centric relief motion. When prompted for feedback after their participation, observers reported experiences of self-rotation in ego-centric conditions, as though they were spinning on a swivel chair. They reported experiences of centrifugal motion in world-centric conditions, as though they were being flung around the 3D object situated in front of them.

Do these differences in the path of self-motion influence vection strength? Figure 2(b) shows mean vection strength ratings following 30 s viewing of the same simulations. A two-way ANOVA found no main effect of fixation, *F*(1, 15) = 0.18, *p* = .68. There was a main effect of relief motion type, *F*(1, 15) = 8.16, *p* < .05, but no interaction effect, *F*(1, 15) = 0.43, *p* = .52. The vection and perceived axis of rotation data together point to the possibility that vection might depend on the inferred distance of axis of rotation; perceiving more centrifugal self-motion leads to stronger experiences of vection, compared with the perception of on-axis self-rotation. If this notion is at the very least supported by the significant differences above, then we should find an observer-wise relationship between vection and perceived axis of rotation.

To test this idea, we performed a correlation between the normalized estimates of vection strength and perceived axis of rotation. Data across fixation conditions were averaged for each observer. Figure 3 plots normalized vection strength as a function of normalized perceived axis of rotation in depth. Each data point corresponds to the average across conditions with and without fixation. In support of the view that vection is influenced by perceived axis of self-rotation, there was a significant correlation between vection strength and perceived axis of rotation in depth, *r* = +0.56, *t*(30) = 3.75, *p* < .001. This relationship suggests that approximately 31% of the variability in vection strength estimates is explainable by variations in the perceived axis of self-rotation.

**Figure 3.**
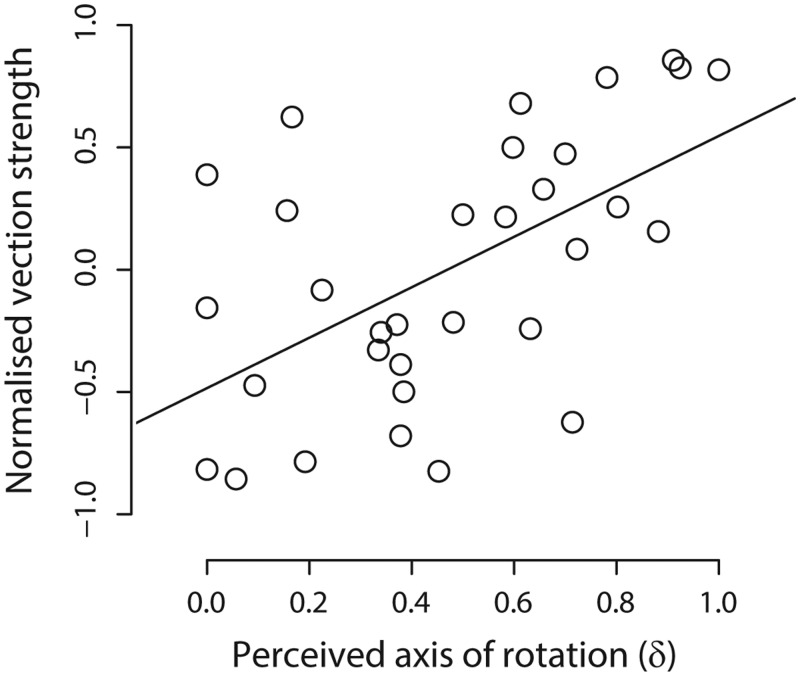
Normalized vection strength estimates plotted as a function of perceived axis of (self-)rotation in depth. Line of best fit superimposed. Values for perceived axis location in depth range from 0.0 (observer-centred) to 1.0 (object-centred). Note the positive relationship between vection strength and perceived distance of the axis of rotation in depth (*r* = +0.56).

These findings extend those of a previous study by Nakamura and Shimojo (1999) who found that vection generated by background motion could be increased by adding slow foreground motion in the opposite direction. Our data replicate this finding in circular vection induced by simulated self-rotation and further suggest this effect can be explained by differences in the perceived axis of rotation.

Seno et al. (2009) showed that vection is generated by motion perceived as the background, leading to their support for “the object and background hypothesis in vection.” However, we placed a foreground object in all our conditions, so the light field would have generated experiences of background motion unambiguously across conditions. Also, the perceived distance of the foreground object did not vary significantly between experiences of world-centred and object-centred motion due to the available disparity cues. Hence, the effects we observe are consistent with vection depending not on background motion per se, but on dynamic properties of perceived scene layout.

To informally verify that the direction of foreground motion relative to the background was critical for vection effects observed here, we presented displays with inverted object rotation to three of the observers. All reported that vection generated by these inverse-rotation displays was weaker than that generated by the world-centric rotation condition. Observers indicated after viewing these displays that the condition with the inversely rotating object appeared unnatural or even plain “weird” (quoting one observer), as though the object was moving independently. This suggests that vection strength depends on the precise configuration of foreground–background motions in the display, and not merely the motion of the foreground object per se. It is possible that this decline in vection might be driven by a decline in the realistic appearance of the display (Riecke, Schulte-Pelkum, Avraamides, Heyde, & Bülthoff, 2006) or the inability to compute a valid interpretation of the scene’s configuration (Ogawa & Seno, 2014; Seno & Fukuda, 2012).

The pattern of data we obtained is also consistent with the view that vection is not impaired by sensory conflicts. Contrary to Zacharias and Young (1981) who proposed that vection should be optimized when sensory conflict is minimized, we find greater vection in conditions of apparent off-axis self-rotation expected to apply centrifugal forces to the head. This expected stimulation should increase greater activity of otolithic vestibular receptors, compared with perceiving on-center rotation. However, the resulting sensory conflict increased vection strength in our stationary observers, rather than impairing it. This finding supports other research showing that vection is increased by viewpoint jitter, which is expected to generate greater otolithic stimulation and sensory conflicts (Palmisano et al., 2000).

Observer reports of the perceived axis of rotation varied significantly across conditions with or without relative object rotation. This variation in their inferred axis of self-rotation accounted for nearly one-third of the variation in normalized vection strength ratings. Although engagement of eye movements can influence vection strength (Kim & Palmisano, 2010), it is possible that there may have been little engagement of eye movements in free viewing conditions, as we found little difference compared with fixation conditions. Hence, any potential influence of retinal motion on this effect is yet to be identified.

Together, the results of the current study support the view that the strength of vection and perceived path of traversal depends on mid-level interpretations of foreground object motion in the context of background motion. The novel findings with the perceived axis of rotation could be extended in future research to investigate the role of different forms of perceptual scene decomposition on the experience of self-motion in real-world contexts.

## Methods

### Observers

Sixteen adult observers with normal or corrected-to-normal visual acuity participated in the study. Observer recruitment and participation in the study adhered to ethical principles of the Human Research Ethics Advisory panel (HREA) for biomedical research at the University of New South Wales and the Declaration of Helsinki.

### Stimulus Generation and Display

The scene was a background illumination map, the Uffizi light field, used commonly in graphical lighting simulations (Debevec, 2002). This background was simulated at infinity. The foreground object was a bumpy spheroid generated by perturbing the 10,250 vertices of a geodesic sphere using cloud-noise as a height map. The graphical simulation was generated by custom software written in C/C++ with embedded calls to the OpenGL Shading Language. The object occupied approximately ±15° of the central visual field, and it was simulated at a distance of 2 m from the observer.

The display of scene content was administered using the Oculus Rift Development Kit 2. The simulation made calls to the Rift’s development library to present stereo images of the world with disparity to each eye. Hence, each frame in time was rendered twice, once for the left and once for the right eye’s view, achieving approximately 60 fps for each eye. The light field was rotated around the object and observer in yaw at approximately 0.125 Hz across all conditions. The display had an approximate horizontal field of view of 110° (80° vertical) with 18 pixels per degree visual angle, comparatively better than the Oculus Rift DK 1 (Kim, Chung, Nakamura, Palmisano, & Khuu, 2015).

### Procedure

There were four conditions in a 2 × 2 design: fixation condition (fixation or no fixation) and object motion (ego-centric or world-centric). Ego-centric rotation was where the image of the object did not change across conditions, consistent with an observer rotating on a swivel chair while inspecting an object they hold in front of them. World-centric rotation was where the image of the object changed to be consistent with the observer walking at a constant radial distance around a completely stationary object.

Observers were exposed to randomized presentations of each display condition for a period of time, after which time, they used a rating bar to set either their report of the vection strength or the apparent location of the axis of self-rotation between the object and themselves. The same rating bar was used to indicate responses over a 0 to 1 range. In vection strength rating conditions, two repeat trials were presented, each for a total of 30 s duration. In conditions where the axis of rotation was estimated, presentations were limited to 10 s and one repeat trial per condition.

Responses were recorded and averaged into mean scores from each observer in each condition. A two-way repeated-measures ANOVA was performed to test for any main or interaction effects. Correlation was used to test for relationships between normalized vection strength and perceived axis of rotation. Vection strengths were normalized for each observer in this correlational analysis to eliminate variability introduced by differences in the psychophysical range of vection experiences across observers (Normalized score = raw/*SD*).
